# DNA Methylation Patterns Can Estimate Nonequivalent Outcomes of Breast Cancer with the Same Receptor Subtypes

**DOI:** 10.1371/journal.pone.0142279

**Published:** 2015-11-09

**Authors:** Min Zhang, Shaojun Zhang, Yanhua Wen, Yihan Wang, Yanjun Wei, Hongbo Liu, Dongwei Zhang, Jianzhong Su, Fang Wang, Yan Zhang

**Affiliations:** 1 College of Bioinformatics Science and Technology, Harbin Medical University, Harbin 150081, China; 2 Department of General Surgery, The Second Affiliated Hospital of Harbin Medical University, Harbin 150086, China; University of North Carolina School of Medicine, UNITED STATES

## Abstract

Breast cancer has various molecular subtypes and displays high heterogeneity. Aberrant DNA methylation is involved in tumor origin, development and progression. Moreover, distinct DNA methylation patterns are associated with specific breast cancer subtypes. We explored DNA methylation patterns in association with gene expression to assess their impact on the prognosis of breast cancer based on Infinium 450K arrays (training set) from The Cancer Genome Atlas (TCGA). The DNA methylation patterns of 12 featured genes that had a high correlation with gene expression were identified through univariate and multivariable Cox proportional hazards models and used to define the methylation risk score (MRS). An improved ability to distinguish the power of the DNA methylation pattern from the 12 featured genes (p = 0.00103) was observed compared with the average methylation levels (p = 0.956) or gene expression (p = 0.909). Furthermore, MRS provided a good prognostic value for breast cancers even when the patients had the same receptor status. We found that ER-, PR- or Her2- samples with high-MRS had the worst 5-year survival rate and overall survival time. An independent test set including 28 patients with death as an outcome was used to test the validity of the MRS of the 12 featured genes; this analysis obtained a prognostic value equivalent to the training set. The predict power was validated through two independent datasets from the GEO database. The DNA methylation pattern is a powerful predictor of breast cancer survival, and can predict outcomes of the same breast cancer molecular subtypes.

## Introduction

Breast cancer is the second largest cause of morbidity worldwide, the first cause of tumors in women [1], and the leading cause of cancer death in women. Moreover, the incidence rates of breast cancer are continuing increase [2]. Breast cancer has multiple molecular subtypes that are classified using tumor biomarkers, such as hormone receptors (HR) (i.e., estrogen receptor (ER) and progesterone receptor (PR)) and human epidermal growth factor receptor 2 (Her2) [3]. Four clinical subtypes of breast cancer can be separated according to HR expression and the epithelial cell of origin (luminal or basal): Luminal A (HR+/Her2-), Luminal B (HR+/Her2+), Her2-enriched (HR-/Her2+), and triple-negative (HR-/Her2-) [4–7]. Breast cancer is a highly heterogeneous disease between and within tumors as well as among cancer-bearing individuals, which is a challenge for the diagnosis, treatment and prognosis [8].

DNA methylation is an epigenetic modification that plays important roles in gene expression regulation, cellular differentiation, development and even tumorigenesis. DNA methylation often occurs at the C-5 position of cytosine, especially cystonsines located in C-phosphate-G (CpG) sites. DNA hypermethylation in gene promoter or CpG islands can result in tumor suppressor silencing, leading to tumorigenesis. Therefore, a large number of differentially methylated regions in cancer have been identified to explore the epigenetic regulation mechanisms underlying oncogenesis [9]. Recently, DNA methylation biomarkers for the diagnosis, molecular typing and prognosis of breast cancer were identified. For example, hypermethylation of RASSF1A can be used to detect breast cancer during the early stages using a CpG island that is hypermethylated in 60–70% of breast cancers [10, 11] or a promoter that is hypermethylated in 70% of breast cancer individuals [12]. Methylated RASSF1A is strongly associated with metastasis, tumor size, and an increased risk of death [13]. BRCA is a well known tumor suppressor for both breast and ovarian cancer whose mutations are more likely to be higher grade, poorly differentiated, highly proliferative, ER negative, PR negative and harbor p53 mutations [14, 15]. However, Xu et al. found that although methylation of the BRCA1 promoter was more prevalent in cancers with tumors size greater than 2 cm, hypermethylation of BRCA1 from breast cancers with BRCA1 mutations had no overall correlation with ER, PR or grade [16]. Aberrant DNA methylation may be correlated with more advanced tumor stages at the time of diagnosis, but it is independent of BRCA1 mutation. Furthermore, DNA methylation has several advantages over sequence mutations as a cancer biomarker [17]. First, the aberrant methylation of specific CpG islands or gene promoters is more frequent than mutations. Second, aberrant methylation patterns can be detected even when they are embedded in an excess amount of normal DNA molecules. Third, techniques for the detection of methylation patterns are relatively simple [18].

Breast cancers can have different treatment responses and overall outcomes even when they are at the same stage of the disease or have the same subtype. Therefore, a good prognostic biomarker of breast cancer can not only contribute to the accurate classification of the subtype but also guide clinical treatment and improve breast cancer outcomes. Signatures predicting the clinical outcomes of breast cancer based on gene expression profiling have been identified and will provide benefits for adjuvant therapy [19]. However, the identification of prognostic predictors in breast cancer that regulate gene expression may have more benefits than unstable gene expression. For example, aberrant methylation of the TSC [20], SFRP1 [21], and RASSF1A [22] genes was associated with an unfavorable prognosis of breast cancer and could be regarded as independent predictors. Although several methylation biomarkers have been identified to predict breast cancer survival, they are usually limited to average methylation levels of several genes based on experiential knowledge. However, there is a weak correlation between the average DNA methylation levels of gene promoter and gene expressions in genome wide [23]. This finding prompted us to hypothesize that methylated CpGs in promoters might be not have equivalent regulatory effects on gene expression. Here, we used canonical correlation analysis to obtain the methylation patterns of CpGs with the strongest correlation with gene expression, and identified predictors of breast cancer prognosis based on high throughput DNA methylation data. The methylated features showed a good distinction of breast cancer outcomes even in samples with the same receptor status.

## Materials and Methods

### Data downloading and processing

DNA methylation data sets of breast cancers based on Human Infinium 450K arrays were obtained from TCGA (http://cancergenome.nih.gov/). Gene expression data sets of breast cancers based on AgilentG4502A_07 were also downloaded from TCGA. Breast cancer samples that had both DNA methylation and gene expression information were retained as the training set. Other samples with only DNA methylation and patient outcome information were regarded as the test set. Samples or CpG sites missing data in the training set were filtered in the following analysis. Additionally, two DNA methylation datasets based on Human Infinium 450K arrays (GSE37754) and Human Infinium 27K arrays (GSE20712) were downloaded from GEO database (http://www.ncbi.nlm.nih.gov/geo/) as independent test sets to assess the predictive power of the DNA methylation biomarkers.

### Canonical correlation analysis

Due to the multiple CpGs located in the gene promoters and the variability of the DNA methylation levels of multiple CpGs located in the same gene, canonical correlation analysis was used to estimate the correlation between gene expression and DNA methylation levels from multiple CpGs. Let $Y^{i}=\{y_{1}^{i},y_{2}^{i},\ldots ,y_{n}^{i}\}$ denote the expression levels of the i*th* gene among *n* samples, and $X^{i}=\{X_{1}^{i},X_{2}^{i},\ldots ,X_{p}^{i}\}$ denote the DNA methylation levels of *p* CpGs from the i*th* gene, where $X_{j}^{i}={\left\{x_{1j}^{i},x_{2j}^{i},\ldots ,x_{nj}^{i}\right\}}^{t}$ is the methylation levels of the j*th* CpG among n samples. For each gene, we can describe the methylation and expression data using the following matrix.

$$D={\left[\begin{array}{ccccc} x_{11} & x_{12} & \cdots & x_{1p} & y_{1}\\ x_{21} & x_{22} & \cdots & x_{2p} & y_{2}\\ \vdots & \vdots & & \vdots & \vdots \\ x_{n1} & x_{n2} & \cdots & x_{np} & y_{n} \end{array}\right]}_{n\times \left(p+1\right)}$$

Assuming the mean and covariance of X and Y were

$$E\left(X\right)=\bar{X}\;\text{and}\;Cov\left(X\right)=\Sigma_{XX}$$

$$E\left(Y\right)=\bar{Y}\;\text{and}\;Cov\left(Y\right)=\Sigma_{YY}$$

The corresponding covariance matrix was Σ.

$$\sum =\left[\begin{array}{cc} \Sigma_{XX} & \Sigma_{XY}\\ \Sigma_{YX} & \Sigma_{YY} \end{array}\right]$$

U and V were the linear combination of *X*_j_ and *Y* respectively.

$$\begin{array}{l} U=\alpha_{1}\cdot X_{1}+\alpha_{2}\cdot X_{2}+\cdots \alpha_{p}\cdot X_{p}=\alpha^{\prime }\cdot X\\ V=\beta \cdot Y \end{array}$$

Thus

$$E\left(U\right)=\alpha^{\prime }\cdot \bar{X}$$

$$E\left(V\right)=\beta \cdot \bar{Y}$$

$$\sum_{UU}=\alpha^{\prime }\sum_{XX}\alpha$$

$$\sum_{UV}=\sum_{VU}=\alpha^{\prime }\sum_{XY}\beta$$

$$\sum_{VV}=\beta \sum_{YY}\beta$$

The objective function that obtained the maximum correlation between U and V was solved based on the Lagrange multiplier method.

$$\{\begin{array}{l} \max\left(r\left(U,V\right)=\frac{\alpha^{\prime }\sum_{XY}\beta }{\sqrt{\alpha^{\prime }\sum_{XX}\alpha }\cdot \sqrt{\beta^{\prime }\sum_{YY}\beta }}\right)\\ \;s.t.\sum_{UU}=\sum_{VV}=1 \end{array}$$

The estimated value $\hat{\alpha }$ and $\hat{\beta }$ constituted the canonical variables U and V that obtained the maximum correlation. Therefore, the methylation pattern of multiple CpGs located in the gene promoter was represented by U which was estimated through the following equation.

$$U={\hat{\alpha }}_{1}\cdot X_{1}+{\hat{\alpha }}_{2}\cdot X_{2}+\cdots {\hat{\alpha }}_{p}\cdot X_{p}$$

The expression pattern was represented by V which was estimated through the following equation.

$$V=\hat{\beta }\cdot Y$$

where $\hat{\beta }$ denoted the regulatory direction of methylation on expression. A $\hat{\beta }$ less than 0 indicated negative regulation; conversely, a $\hat{\beta }$ greater than 0 indicated positive regulation. Additionally, *r*(*U*,*V*) was the correlation degree between the methylation pattern and expression pattern.

For each gene, the methylation pattern score (MPS) was defined as

$$MPS_{k}={\hat{\alpha }}_{1}\cdot x_{k1}+{\hat{\alpha }}_{2}\cdot x_{k2}+\cdots {\hat{\alpha }}_{p}\cdot x_{kp}$$

where, *x*_*kj*_ was the DNA methylation levels of the j*th* CpG in the k*th* sample.

### Survival analysis

The log-rank test was used to identify a subset of genes whose MPS showed significant differences between the high and low groups and to obtain a *p* value. Univariate and multivariable analyses were performed using Cox proportional hazards models incorporating MPS and known prognostic clinical factors, including age at diagnosis (≤55 vs ≥56 years), tumor pathological stage (I & II vs III & IV), and tumor size (1–2 vs 3–4) as categorical variables. Univariate Cox regression analysis was performed to assess the survival prognosis capabilities of the selected gene set using the overall survival time as a dependent variable. To create an optimal feature for genes based on methylation patterns to assess breast cancer outcomes, the methylation risk score (MRS) was defined through featured genes identified based on multivariable Cox proportional hazards models with the MPS as a continuous variable.

$$MRS_{k}=c_{1}\cdot gene_{1k}+\cdots +c_{m}\cdot gene_{mk}$$

where *k* was the k*th* sample. *m* was the number of feature genes filtered by the multivariable Cox proportional hazards models and *c*_j_ (j = 1, 2, …, m) was the coefficient estimated by the multivariable Cox proportional hazards models. The 5-year overall survival for each MRS scoring group (high vs low) was calculated using the Kaplan–Meier method, and the statistical significance was assessed using the log-rank test. The significance level of all statistical tests was 0.05.

### Multiple test correction

To control the false discover rate (FDR) of the featured genes, we adopted two methods to correct the *p* value of the statistical test. For the identification of the gene subset based on MPS through the log-rank test, sample labels were permutated 100 times and the log-rank test was re-performed. Empirical *p* values were calculated according to the order of the observed *p* value among the 100 permutations. If the empirical *p* value was less than 0.01, the gene was retained. Thus, all *p* values from the 100 permutations were larger than the observed *p* value. To obtain the significant candidate gene set with the univariate Cox proportional hazards models, we again permuted the sample labels 100 times. In each permutation, we obtained the *p*-value of the univariate Cox proportional hazards models. According to the FDR equation, the cutoff of *p* (*p*_0_) was determined through FDR = 0.01.

$$FDR=\frac{\frac{1}{n}\cdot \sum_{i=1}^{n}\#{\left(p<p_{0}\right)}_{permutation_{i}}}{\#{\left(p<p_{0}\right)}_{observed}}$$

where the numerator was the expected number of genes whose *p* value from the univariate Cox proportional hazards models was random less than cut off *p*_0_ in random and the denominator was the number of genes whose *p* value was less than *p*_0_ in the real situation.

## Results

### Identification of featured biomarkers based on the effect of DNA methylation regulatory patterns of CpGs on expression

The gene expression and DNA methylation data from 209 breast cancer patients were obtained after processing the missing data from TCGA, which included 15,801 genes and 281,066 CpGs located in gene promoters. The breast cancer details are shown in S1 Table. The methylation patterns of CpGs in gene promoters that showed the maximum correlation with the gene expression patterns were obtained through canonical correlation analysis, which measured the maximum regulatory effect of DNA methylation on gene expression. Pearson's correlation analysis was performed between the average methylation of CpGs and gene expression. However, the canonical correlation degree was significantly higher than the Pearson's correlation degree (Wilcoxon *p* value < 2.2 × 10^−16^, S1A Fig). Moreover, we found that DNA methylation had a negative regulatory effect on the expression of some genes and a positive regulatory effect on others (S1B Fig).

The MPS was calculated through the canonical variable from the canonical correlation analysis to estimate the methylation patterns of CpGs. High- and low-MPS groups were classified based on the median MPS among the samples. The log-rank test was used to identify genes that showed significant difference in outcomes between the high- and low-MPS groups. The *p* value of the log-rank test of these genes was less than 0.05 and the lowest of 100 permutations. A gene subset including 151 genes was identified. Furthermore, 38 genes were retained through univariate Cox proportional hazards models with *p* < 0.05 and FDR = 0.01 among 100 permutations (S2 Table).

The methylation prognostic biomarkers of breast cancer were identified through multivariable Cox proportional hazards models based on 38 genes with p < 0.01. The methylation prognostic biomarkers consisted of 7 protective genes with negative Cox proportional hazard model regression coefficient, indicating that the survival time increased as the MPS increased, and 5 risk genes with positive coefficients, indicating that the MPS increased as the survival time decreased (Table 1).

**Table 1:** Multivariate Cox proportional hazard model of risk gene set.

Gene	Coef	HR	*p* value	95% CI
**PFN1**	-54.0	3.60×10^−24^	0.002025	[4.67×10^−39^, 2.78×10^−9^]
**ZFAND5**	-45.9	1.23×10^−20^	0.002271	**[**2.01×10^−33^, 7.48×10^−8^ **]**
**TMEM184B**	-42.5	3.35×10^−19^	0.008188	**[**6.76×10^−33^, 1.66×10^−5^ **]**
**PPP1R12C**	-41.2	1.24×10^−18^	0.000633	**[**6.64×10^−29^, 2.31×10^−8^ **]**
**NRIP2**	-40.6	2.45×10^−18^	0.000455	**[**3.50×10^−28^, 1.71×10^−8^ **]**
**DPAGT1**	-34.2	1.36×10^−15^	0.001741	**[**6.71×10^−25^, 2.75×10^−6^ **]**
**HPX**	-29.2	2.06×10^−13^	0.002975	**[**8.77×10^−22^, 4.84×10^−5^ **]**
**HERPUD2**	27.9	1.32×10^12^	0.009686	**[**8.65×10^2^, 2.01×10^21^ **]**
**ZNF592**	30.5	1.83×10^13^	0.00128	**[**1.55×10^5^, 2.17×10^21^ **]**
**ZNF536**	51.4	2.03×10^22^	0.000563	**[**4.27×10^9^, 9.60×10^34^ **]**
**SLC25A21**	51.6	2.52×10^22^	0.008259	**[**6.01×10^5^, 1.06×10^39^ **]**
**NARS**	53.3	1.44×10^23^	0.004414	**[**1.64×10^7^, 1.26×10^39^ **]**

Abbreviations: Coef is the Cox proportional hazard model regression coefficient. HR: hazard ratio; CI: confidence interval; p value: cox regression model p value.

### Estimating breast cancer outcomes according to the methylation risk score

We assessed the MRS of each sample based on the 12 methylation biomarkers described as follows to predict the breast cancer outcomes.

$$\begin{array}{l} MRS=51.36\cdot ZNF536+53.32\cdot NARS+30.54\cdot ZNF592+51.58\cdot SLC25A21+27.91\cdot HERPUD2\\ -53.98\cdot PFN1-45.85\cdot ZFAND5-40.55\cdot NRIP2-29.21\cdot HPX-34.23\cdot DPAGT1\\ -42.54\cdot TMEM184B-41.23\cdot PPP1E12C \end{array}$$

**Fig 1:**
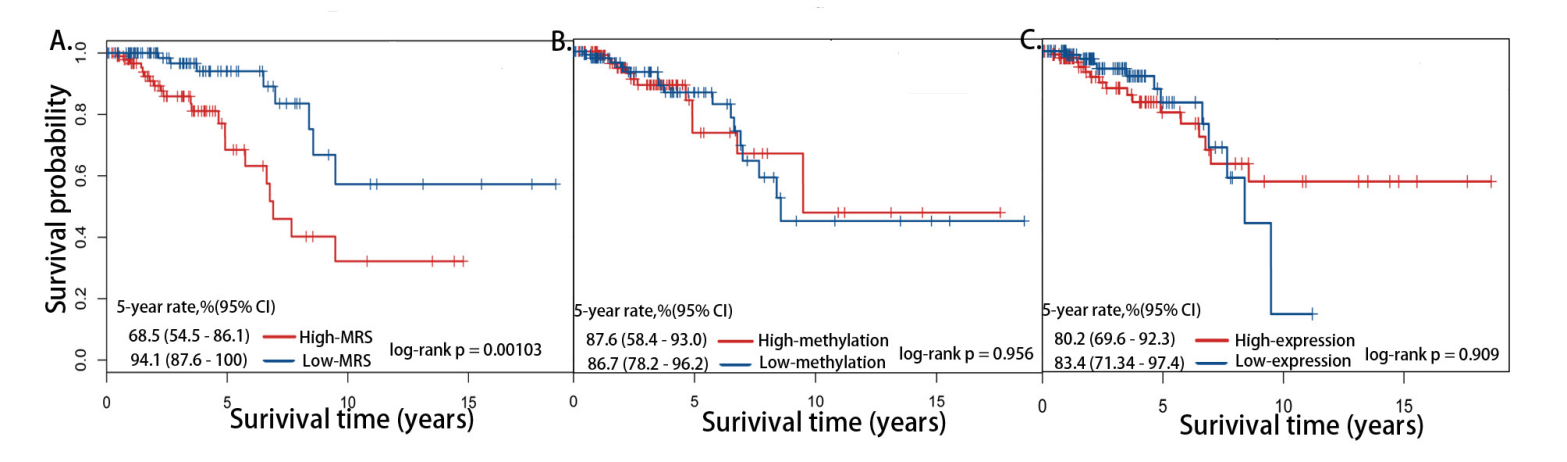
Kaplan-Meier survival analysis of overall survival of 209 breast patients based on feature genes. (A) MRS. (B) Average DNA methylation levels. (C) Average gene expression levels.

The Kaplan–Meier method was used to estimate the 5-year overall survival rate between the high- and low-MRS groups classified through the median MRS among the samples. As shown in Fig 1A, the high-MRS group had a significantly shorter 5-year overall survival rate than the low-MRS group (68.5% vs 94.1%, log-rank *p* = 0.00103). The average methylation and expression levels of 12 featured genes were adopted to perform survival analysis. No significant difference were detected between the high and low groups classified through the median methylation or expression levels (Fig 1B and 1C). Additionally, we used the 7 protective and 5 risk genes for the survival analysis. The overall survival time and 5-year survival rates were significant different between the high and low groups; the 7 protective genes especially allowed more distinct estimations (S2 Fig).

MRS had a significant association with hazard ratio (HR) of death in both the univariate and multivariable Cox proportional hazards models compared with the prognostic clinical factors, including age at diagnosis (≤55 vs ≥56 years), tumor pathological stage (I & II vs III & IV), and TNM (1–2 vs 3–4) (S3 Table). This result suggested that the regulatory effect of the DNA methylation pattern on expression might be better able to predict the outcome of breast cancer than depending on methylation or expression alone.

### Differential outcomes of receptor states with differential MRS

**Fig 2:**
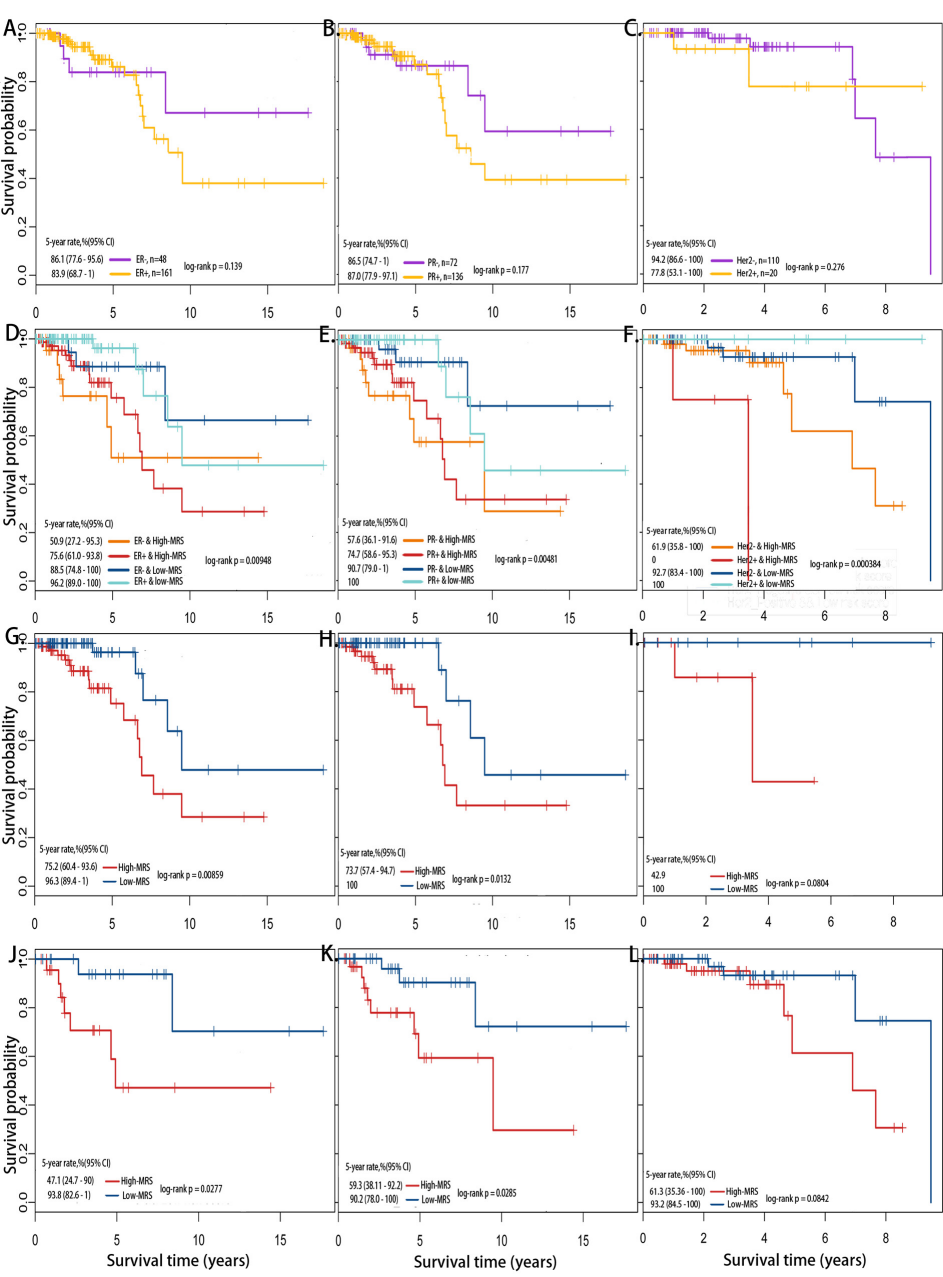
Kaplan-Meier survival analysis of overall survival for receptor status. (A) Survival comparison between ER+ and ER- patients. (B) Survival comparison between PR+ and PR- patients. (C) Survival comparison between Her2+ and Her2- patients. (D) Survival comparison through combination of ER states and MRS. (E) Survival comparison through combination of PR states and MRS. (F) Survival comparison through combination of Her2 states and MRS. (G) Survival comparison between high-MRS and low-MRS from ER+. (H) Survival comparison between high-MRS and low-MRS from PR+. (I) Survival comparison between high-MRS and low-MRS groups from Her2+. (J) Survival comparison between high-MRS and low-MRS groups from ER-. (K) Survival comparison between high-MRS and low-MRS groups from PR-. (L) Survival comparison between high-MRS and low-MRS groups from Her2-.

High heterogeneity and differential outcomes of breast cancer were found among patients from the same subtype or receptor status. However, no significant differences in the overall survival time and 5-year survival rate were found between ER+ and ER-, PR+ and PR-, Her2+ and Her2- patients (Fig 2A–2C). When we combined these factors with the MRS, found a significant difference between the positive and negative receptor status and the breast cancer outcome. For example, ER- & high-MRS samples resulted in the lowest 5-year survival rat, whereas ER+ & low-MRS samples had the highest 5-year survival rate (Fig 2D). Similarly, PR- & high-MRS and Her2- & high-MRS samples exhibited the lowest 5-year survival rates, whereas PR+ & low-MRS and Her2+ & low-MRS samples reached the highest 5-year survival rates (Fig 2E and 2F). Significantly differential outcomes were observed between the high- and low-MRS groups, although the patients had the same receptor state (Fig 2G–2L). In conclusion, differential outcomes were observed when the MRS and receptor status were combined, even when the patients had the same receptor state. We found that ER-, PR- or Her2- patients with high-MRS had the worst outcomes, which was consistent with the known conclusion.

### Application of the methylation risk feature on breast cancers that resulted in death state and independent test datasets

**Fig 3:**
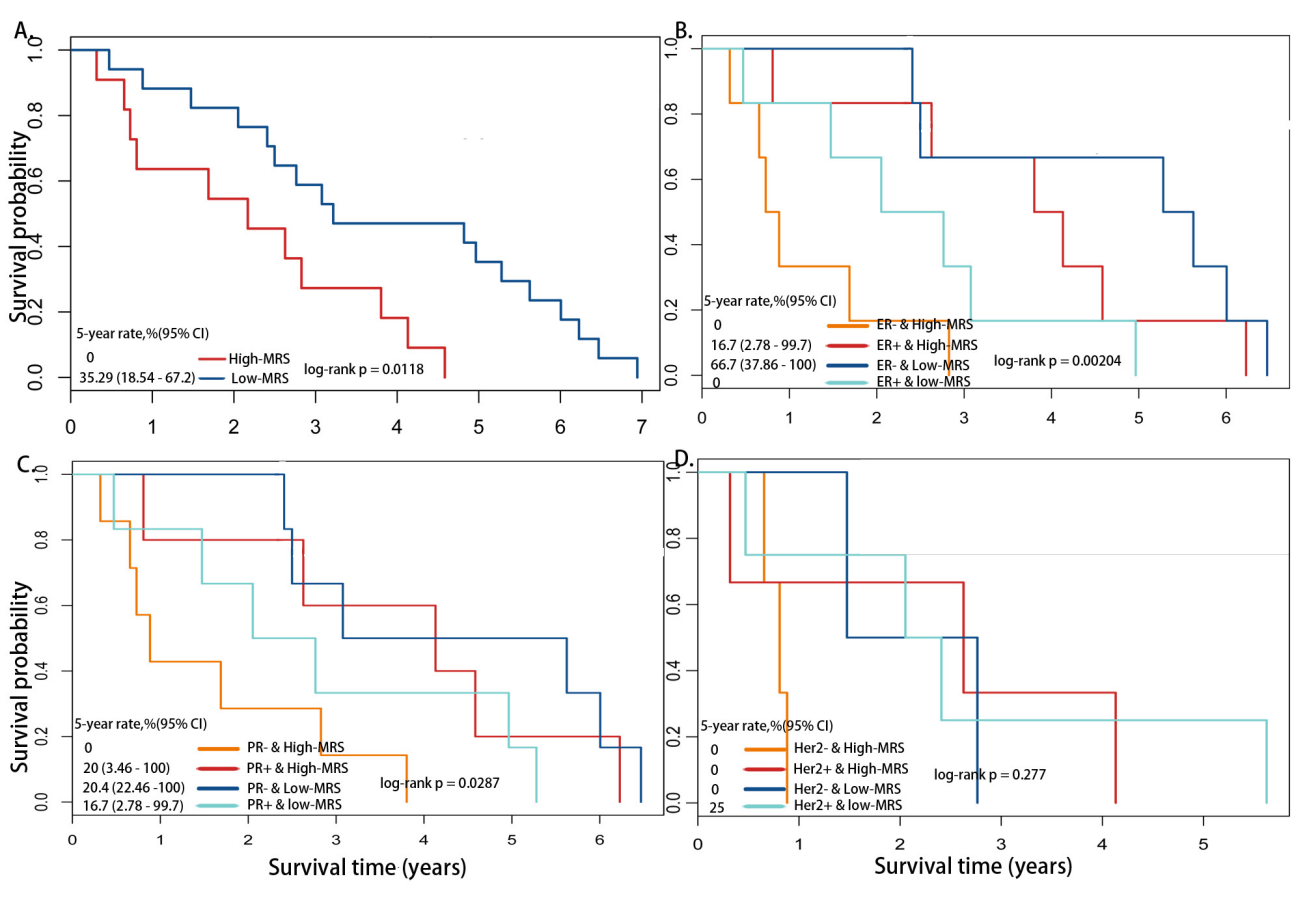
Kaplan-Meier survival analysis of overall survival on 28 patients with death outcome. (A) Survival comparison between High-MRS and Low-MRS groups. (B) Survival comparison among ER+/- patients. (C) Survival comparison among PR+/- patients. (D) Survival comparison among Her2+/- patients.

To validate the prognostic value of DNA methylation biomarkers on other breast samples, the MRS of 12 featured genes were applied to 28 breast samples from patients with a death outcome from the TCGA dataset that only had DNA methylation information and were not included in the previous dataset. We found a significantly differential survival time between the low- and high-MRS groups (Fig 3A). Moreover, the overall survival time of the high-MRS group was less than 5 years. MRS showed a good ability to distinguish outcomes between ER+ and ER-, PR+ and PR-, and Her2+ and Her2- samples (Fig 3B–3D). We observed similar results with the training set, with ER-, PR- or Her2- samples with high-MRS showing the worst prognosis; indeed, less than one year of survival was estimated for the Her2- & high-MRS group. The effect of the prognostic outcome in the independent samples suggested that a DNA methylation biomarker identified through the regulation of gene expression by DNA methylation patterns in CpGs, is believable and has good predictive value for breast cancer outcomes.

**Fig 4:**
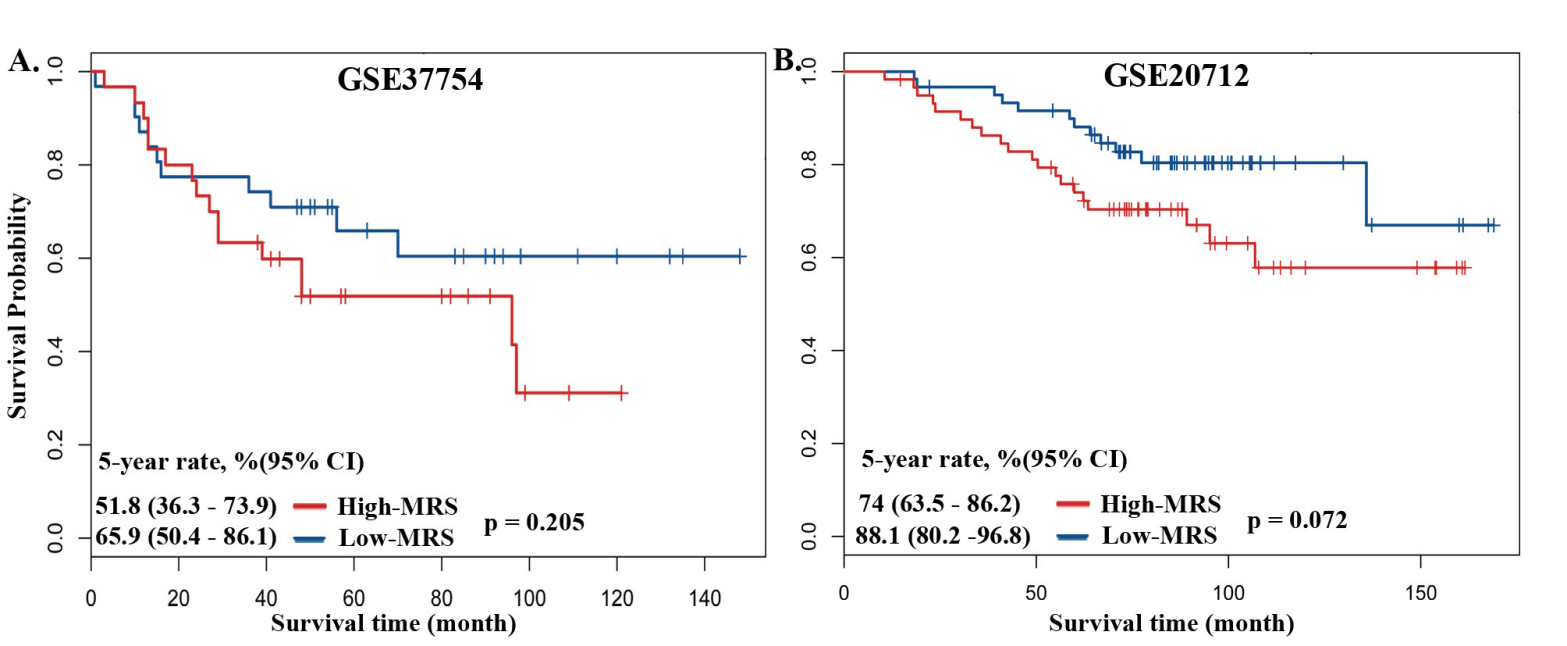
The Kaplan-Meier survival analysis of overall survival on four independent dataset from GEO database. (A) GSE37754 from 450K arrays. (B) GSE20712 from 27K arrays.

Additionally, we evaluated the performance of 12 featured genes using two independent breast cancer datasets (GSE37754 and GSE20712). NARS and SLC25A21 were not included in the 27K DNA methylation dataset (GSE20712), and an additional 10 featured genes were used. All 12 featured genes were used in the 450K dataset (GSE37754). Using two independent cohorts, we found differential outcomes between the high- and low-MRS groups (Fig 4); p values of the log-rank test were especially significant in GSE20712 (Fig 4B). MRS showed a preferable distinguishing power between the high- and low-MRS groups. This finding suggested that DNA methylation biomarkers might be robust factors for the prediction of breast cancer outcomes.

## Discussion

Breast cancer is a heterogeneous tumor. Molecular classification has been successfully used to design individualized therapies, leading to significant improvements in disease-specific survival [24]. Recently, breast cancer was classified into three major subtypes based on luminal, Her2+ and basal-like based gene expression profiling [25, 26]. Moreover, DNA methylation showed distinct patterns among subtypes of breast cancer (especially between luminal B and basal-like) [27]. Each of the breast cancer subtypes has different risk factors for incidence, response to treatment, risk of disease progression and outcomes. For example, triple negative breast cancer which usually includes basal-like tumors that lack HR and Her2, has a worse outcomes than the other subtypes because no specific molecular targets have been identified [28]. Recently, the identification of differential methylated regions of triple-negative breast cancer based on whole-genome methylation sequencing has provided diagnostic and prognostic value for personalized management [29].

We identified DNA methylation patterns of CpGs located within gene promoters that had a maximal regulatory effect on gene expression based on canonical correlation analysis to define the methylation pattern score of adjacent CpGs. DNA methylation featured genes associated with breast cancer outcomes that were obtained according to the DNA methylation patterns, which contributed to the construction of the methylation risk score. The methylation risk score of the featured genes showed the best ability to estimate the survival time between the high and low-risk groups compared to average DNA methylation or gene expression. Moreover, we found significant differential outcomes between the high- and low-MRS groups even though the breast samples had the same HR or Her2 status (especially ER-, PR- or Her2-), with the high-MRS group having the worst outcomes. A similar conclusion was supported using 28 breast samples with death as an outcome. We evaluated the estimation ability of the DNA methylation pattern of featured genes in other breast cancers based on Human Infinium 450K and 27K arrays from the GEO database. Due to the absence of some CpGs and genes in the 27K arrays compared with the 450K arrays, we used shared featured genes and CpGs between the 450K and 27K datasets to measure the MRS. Only 10 featured genes were found (excluding NARS and SLC25A21) in the 27K datasets; these genes were used to predict breast cancer outcomes. We also found differential outcomes between the high- and low-MRS groups in two independent cohorts. However, the p values of the log-rank test from the 450K test sets were not significant. We speculate that the high heterogeneity in cancer may lead to differential outcomes. Notably, the breast samples from GSE37754 (450K arrays) were in the early stages of cancer with tumor pathological stages I or II, which might have caused the non-significant p-value. Although differences in the platform between the 27K and 450K arrays led to the absence of some featured genes and CpGs, the p value of the log-rank test was significant and the breast cancer outcomes were clearly distinguished between the high- and low-MRS groups. This finding implied that the survival model based on the methylation patterns of featured genes showed a good survival prognostic capability in breast cancer.

The DNA methylation featured genes we identified were ZNF536, ZNF592, NARS, SLC25A21, HERPUD2, NRIP2, PPP1R12C, DPAGT1, PFN1, ZFAND5, HPX and TMEM184B. ZNF536, ZNF592 and ZFAND5 were zinc finger proteins that had important roles in transcript regulation, embryonic development and cell differentiation. Several studies reported that SLC25A21 [30], PFN1 [31], HPX [32]and TMEM184B [33] were associated with a risk of breast cancer. Additionally, other genes were associated with the risk of other diseases, such as NARS, which causes nonsyndromic hearing loss, Leigh syndrome [34] and Alpers syndrome [35], and DPAGT1, which is involved in the pathogenesis of oral cancer [36]. The methylation featured genes did not include the BRCA gene. This finding was consistent with the conclusion of Xu et al, who reported that the methylation of BRCA had no overall correlation with ER, PR or grade. These results suggest that DNA methylation is an independent predictor of breast cancer prognosis and is independent of BRCA1 mutation. We attempted to compare the survival time between the BRCA mutation and non-mutation samples through MRS, but the analysis was limited by the small sample number with BRCA mutations. The good prognostic power of DNA methylation biomarkers can help guide clinical treatment and predict the outcome of breast cancer.

## Supporting Information

S1 Fig Feature of correlation between DNA methylation pattern and gene expression based on canonical correlation analysis. (A) Comparison of canonical and pearson's correlation coefficient. (A) Regulatory effect of DNA methylation pattern on gene expression based on canonical correlation analysis.
(TIF)

S2 Fig Kaplan-Meier survival analysis of overall survival of 209 breast patients. (A) Protected genes. (B) Risk genes.
(TIF)

S1 Table Information of Breast invasive carcinome patients from TCGA. (DOC)

S2 Table Results of univariate Cox proportional hazard model about significant gene set. (DOC)

S3 Table Cox proportional hazards analyses using different predictors. (DOC)
