# Supplementary figures and images for: DNA Methylation Patterns Can Estimate Nonequivalent Outcomes of Breast Cancer with the Same Receptor Subtypes
Source: PLoS One. 2015 Nov 9;10(11):e0142279. doi: 10.1371/journal.pone.0142279 (PMC4638352; doi:10.1371/journal.pone.0142279)

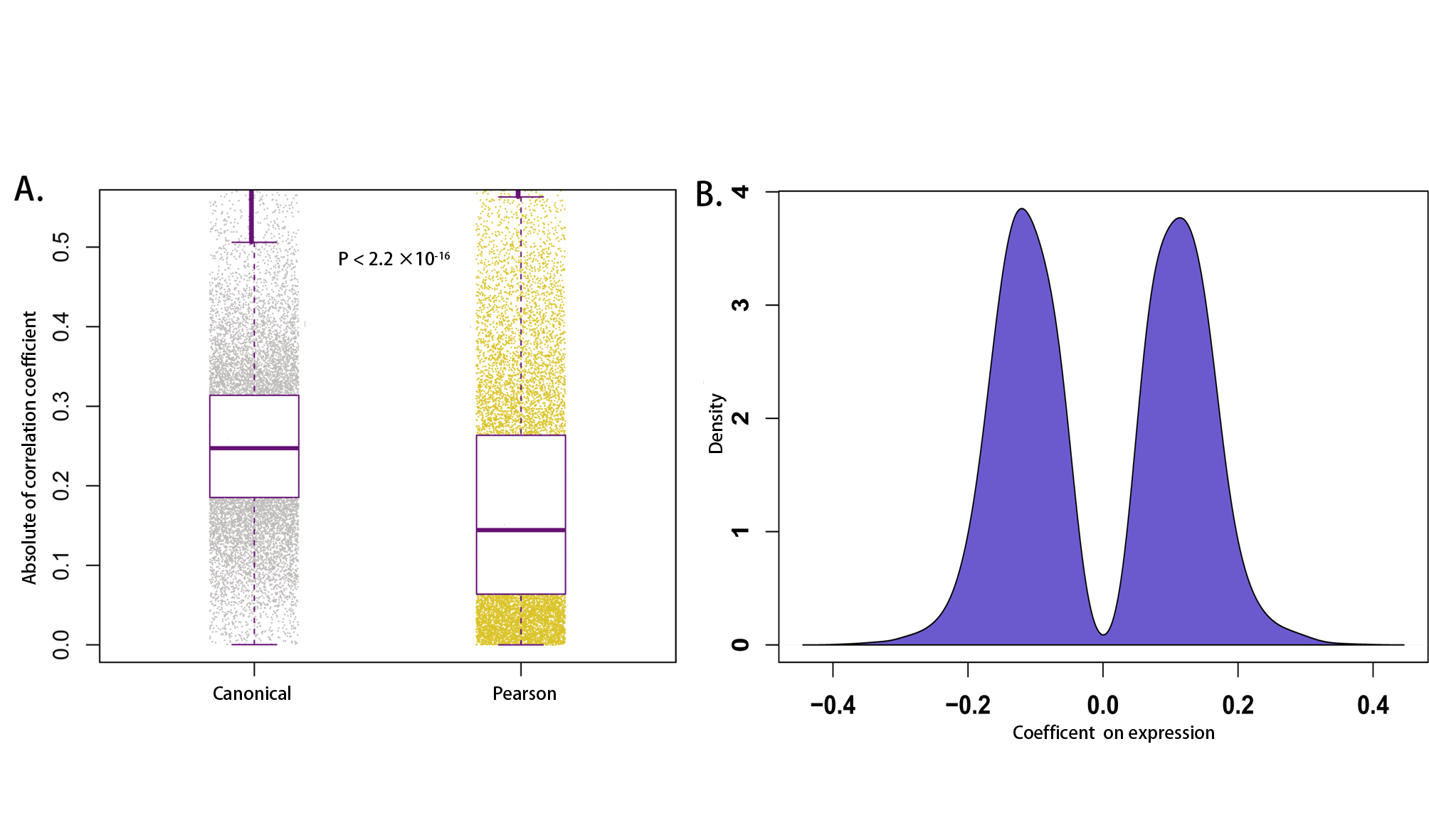

Supplement: S1 Fig — (A) Comparison of canonical and pearson's correlation coefficient. (A) Regulatory effect of DNA methylation pattern on gene expression based on canonical correlation analysis. (TIF) [file pone.0142279.s001.tif]

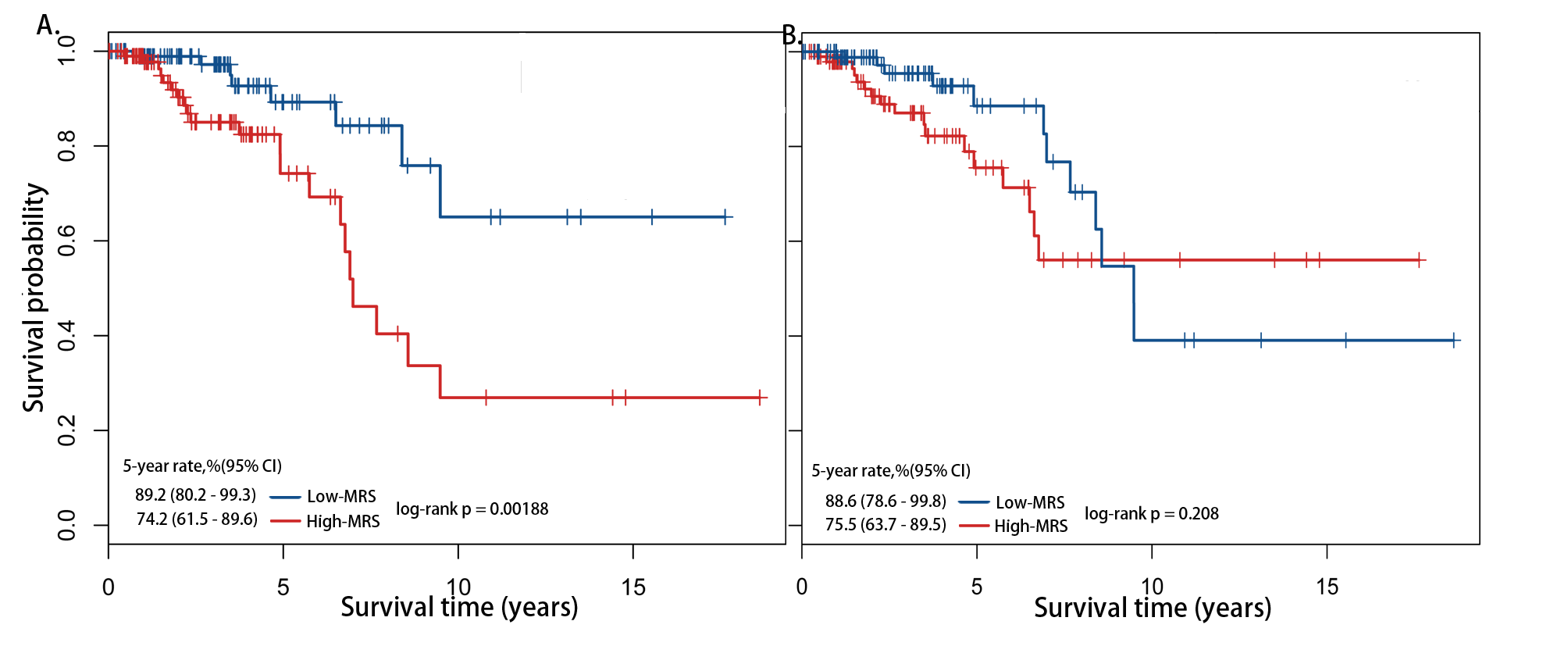

Supplement: S2 Fig — (A) Protected genes. (B) Risk genes. (TIF) [file pone.0142279.s002.tif]
