# Supplementary material for: DNA Methylation Patterns Can Estimate Nonequivalent Outcomes of Breast Cancer with the Same Receptor Subtypes
Source: PLoS One. 2015 Nov 9;10(11):e0142279. doi: 10.1371/journal.pone.0142279 (PMC4638352; doi:10.1371/journal.pone.0142279)
Supplement: S1 Table — (DOC) [file pone.0142279.s003.doc]

Supplementary Table 1. Information of Breast invasive carcinome patients from TCGA

|  | Training set | Test set |
| --- | --- | --- |
| Patient | 209 | 28 |
| Age | | |
| Media | 56 | 66 |
| range | 26~83 | 31~90 |
| <=55 | 103 | 9 |
| >=56 | 106 | 19 |
| TNM | | |
| 1&2 | 142 | 16 |
| 3&4 | 67 | 12 |
| Stage | | |
| Ⅰ&Ⅱ | 141 | 16 |
| Ⅲ&Ⅳ | 68 | 12 |
| Sex | | |
| Female | 209 | 28 |
| Male | 0 | 0 |
| ER Receptor | | |
| Positive | 161 | 10 |
| Negative | 48 | 13 |
| PR receptor | | |
| Positive | 136 | 10 |
| Negative | 72 | 13 |
| Her2 Receptor | | |
| Positive | 20 | 7 |
| Negative | 110 | 5 |

Stage: tumor pathological stage. TNM: tumor size.
