# Supplementary material for: DNA Methylation Patterns Can Estimate Nonequivalent Outcomes of Breast Cancer with the Same Receptor Subtypes
Source: PLoS One. 2015 Nov 9;10(11):e0142279. doi: 10.1371/journal.pone.0142279 (PMC4638352; doi:10.1371/journal.pone.0142279)
Supplement: S2 Table — (DOC) [file pone.0142279.s004.doc]

Supplementary Table2. Results of univariate Cox proportional hazard model about significant gene set

| Gene | Coef | HR | p-value | 95% CI |
| --- | --- | --- | --- | --- |
| RNASE4 | -12.1 | 5.39×10^-6^ | 0.000936 | 4.083×10^-9^ 7.11×10^-3^ |
| ZFAND5 | -11.3 | 1.28×10^-5^ | 0.000549 | 2.138×10^-8^ 7.614×10^-3^ |
| TMEM184B | -10.1 | 4.24×10^-5^ | 0.00142 | 8.758×10^-8^ 2.056×10^-2^ |
| MRPL4 | -10.0 | 4.40×10^-5^ | 0.0000498 | 3.461×10^-7^ 5.603×10^-3^ |
| PRC1 | -9.75 | 5.86×10^-5^ | 0.0000740 | 4.728×10^-7^ 7.257×10^-3^ |
| ALKBH2 | -9.57 | 6.96×10^-5^ | 0.0000335 | 7.551×10^-7^  6.41×10^-3^ |
| ESRRA | -8.7871231 | 1.527×10^-4^ | 0.00403 | 3.828×10^-7^ 6.09×10^-2^ |
| HARS | -8.5061136 | 2.022×10^-4^ | 0.00216 | 8.826×10^-7^ 4.633×10^-2^ |
| MAP3K9 | -8.337193 | 2.394×10^-4^ | 0.000555 | 2.108×10^-6^ 2.72×10^-2^ |
| PDE6H | -8.200628 | 2.745×10^-4^ | 0.000993 | 2.082×10^-6^ 3.619×10^-2^ |
| POM121 | -8.1029834 | 3.026×10^-4^ | 0.00222 | 1.685×10^-6^ 5.435×10^-2^ |
| TRIP12 | -7.9676219 | 3.465×10^-4^ | 0.00332 | 1.697×10^-6^ 7.073×10^-2^ |
| CDKL2 | -7.4732229 | 5.681×10^-4^ | 0.00147 | 5.674×10^-6^ 5.687×10^-2^ |
| NRIP2 | -7.4561316 | 5.779×10^-4^ | 0.00126 | 6.223×10^-6^ 5.366×10^-2^ |
| DCLRE1C | -7.409354 | 6.056×10^-4^ | 0.00317 | 4.41×10^-6^ 8.315×10^-2^ |
| HPX | -7.2162851 | 7.345×10^-4^ | 0.00176 | 7.979×10^-6^ 6.762×10^-2^ |
| DPAGT1 | -7.1789349 | 7.625×10^-4^ | 0.00436 | 5.479×10^-6^ 0.1061 |
| DIAPH1 | -7.1386879 | 7.938×10^-4^ | 0.00243 | 7.856×10^-6^ 8.021×10^-2^ |
| C11orf82 | -7.0694075 | 8.507×10^-4^ | 0.00314 | 7.803×10^-6^ 9.275×10^-2^ |
| PPP3CA | -6.805068 | 1.108×10^-3^ | 0.00142 | 1.696×10^-5^ 7.24×10^-2^ |
| NARS | -6.799762 | 1.114×10^-3^ | 0.00158 | 1.641×10^-5^ 7.564×10^-2^ |
| SLC25A16 | -6.53633 | 1.45×10^-3^ | 0.00251 | 2.089×10^-5^ 1.006×10^-1^ |
| PPP1R12C | 6.343 | 5.68773×10^2^ | 0.00233 | 9.577 3.3779×10^4^ |
| HERPUD2 | 6.693 | 8.06732×10^2^ | 0.00846 | 5.534 1.17607×10^5^ |
| FBXO16 | 7.34 | 1.540174×10^3^ | 0.000777 | 21.32 1.11273×10^5^ |
| ZNF592 | 7.399 | 1.63388×10^3^ | 0.00181 | 15.66 1.70440×10^5^ |
| DNAJB11 | 7.484 | 1.780.048×10^3^ | 0.00128 | 18.74 1.69124×10^5^ |
| ENSA | 7.49 | 1.789.848×10^3^ | 0.003 | 12.73 2.51632×10^5^ |
| SLC25A21 | 7.997 | 2.971.917×10^3^ | 0.00341 | 14.07 6.27833×10^5^ |
| ZNF536 | 8.296 | 4.009.681×10^3^ | 0.00149 | 23.95 6.71176×10^5^ |
| TMEM9 | 8.374 | 4.334.811×10^3^ | 0.000826 | 32.01 5.87012×10^5^ |
| LHFPL3 | 8.446 | 4.655.193×10^3^ | 0.00182 | 23.04 9.40701×10^5^ |
| HS6ST2 | 8.568 | 5.261.376×10^3^ | 0.000782 | 35.47 7.80401×10^5^ |
| ACAA1 | 9.002 | 8.116.063×10^3^ | 0.000252 | 65.42 1.006900×10^6^ |
| MGST3 | 9.276 | 1.0676714×10^4^ | 0.00719 | 12.33 9.244009×10^6^ |
| PSMC3 | 9.952 | 2.0990548×10^4^ | 0.000641 | 69.29 6.358937×10^6^ |
| PFN1 | 11.6 | 1.10×10^5^ | 0.000536 | 1.537×10^2^ 7.8124451×10^7^ |
| ANP32C | 13.9 | 1.12×10^6^ | 0.0000166 | 1.974×10^3^ 6.30694668×10^7^ |

Abbreviations: Coef is the Cox proportional hazard model regression coefficient. HR : hazard ratio. CI: confidence interval. P-value: cox regression model p value.
