# Supplementary material for: DNA Methylation Patterns Can Estimate Nonequivalent Outcomes of Breast Cancer with the Same Receptor Subtypes
Source: PLoS One. 2015 Nov 9;10(11):e0142279. doi: 10.1371/journal.pone.0142279 (PMC4638352; doi:10.1371/journal.pone.0142279)
Supplement: S3 Table — (DOC) [file pone.0142279.s005.doc]

**S**upplementary Table 3. Cox proportional hazards analyses using different predictors

| Factors | Univariate | | | Multivariable | | |
| --- | --- | --- | --- | --- | --- | --- |
|  | HR | P-value | 95% CI | HR | P-value | 95%CI |
| MRS | 1.12 | 1.91×10^-6^ | 1.07 -1.17 | 1.12 | 5.28×10^-6^ | 1.07 - 1.17 |
| Age | 1.03 | 0.087 | 1.00 - 1.06 | 1.01 | 0.46 | 0.98 - 1.04 |
| TNM (1,2) vs (3,4) | 1.14 | 0.51 | 0.78 - 1.67 | 0.95 | 0.84 | 0.55 - 1.62 |
| Stage (Ⅰ,Ⅱ) vs (Ⅲ,Ⅳ) | 1.65 | 0.19 | 0.79 - 3.45 | 1.86 | 0.25 | 0.65 - 5.32 |
